# Approach to biliary tree clearance in pediatric patients undergoing cholecystectomy: insights from a tertiary hospital

**DOI:** 10.1007/s00383-025-06037-3

**Published:** 2025-05-20

**Authors:** Tal Weiss, Yael Dreznik, Maya Paran, Dragan Kravarusic

**Affiliations:** 1 Department of Pediatric and Adolescent Surgery, Schneider Children’s Medical Center of Israel, Petah-Tiqwa, Israel; 2 Faculty of Medicine, Tel Aviv University, Kiryat HaUniversita, Ramat Aviv, Tel Aviv, Israel; 3 Department of General Surgery, Hillel Yaffe Medical Center, The Rapaport School of Medicine, Technion, Haifa, Israel

**Keywords:** Pediatric cholecystectomy, CBD stone predictors, Biliary tree clearance

## Abstract

**Introduction:**

Despite the increasing rate of cholecystectomy in pediatric patients, no standardized protocols for perioperative biliary tree clearance in children exist and the applicability of adult guidelines to pediatric patients remains uncertain.

**Aim:**

To identify predictors for CBD stones in pediatric patients undergoing cholecystectomy and to evaluate the applicability of adult guidelines for children.

**Materials and methods:**

We conducted a retrospective study on pediatric patients who underwent cholecystectomy for cholelithiasis at a tertiary pediatric medical center from 2011 to 2024. Medical records were reviewed for demographic and clinical characteristics. Elevated bilirubin was defined as above 4 mg/dL with > 20% conjugated. The outcomes measured included the presence of CBD stones detected by ERCP or intraoperative cholangiography and post-cholecystectomy complications due to retained stones.

**Results:**

A total of 177 patients were included in the study, with a median age of 13.4 years (IQR 9, 16.4). Sixteen patients (9%) were diagnosed with CBD stones. Elevated bilirubin, dilated CBD, and filling defects on primary imaging were strongly associated with CBD stones (50.0% vs. 9.9%, *p* < 0.001, 62.5% vs. 9.3%, *p* < 0.001, 43.8% vs. 4.4%, *p* < 0.001). The 2019 ASGE guidelines had a sensitivity of 56.2% and a specificity of 91.1% for predicting CBD stones. Adjusting the guidelines to classify elevated bilirubin as an independent high-risk feature improved sensitivity to 68.8%, with a slight reduction in specificity to 87.6%.

**Conclusion:**

Our study suggests that the 2019 ASGE guidelines are applicable to children. Based on our findings and previous data, it seems reasonable to classify bilirubin elevation as an independent high-risk feature.

## Introduction

The prevalence of biliary disease and subsequent cholecystectomies in the pediatric population is increasing [1, 2]. This increase can be explained in part by the rise of pediatric obesity, which is associated with higher rates of cholesterol biliary stones [2–5].

In the setting of cholelithiasis, it is customary to assess patients’ risk for common bile duct (CBD) stones prior to surgery in order to avoid post cholecystectomy complications due to retained biliary stones. When common bile duct stones (CBD stones) are suspected, perioperative biliary tree clearance is recommended [6]. The incidence of CBD stones among children with symptomatic gallstones is described to be up to 30% [7, 8], compared to 10–20% in adults [9]. However, biliary tree evaluation in children mostly relies on adult guidelines, with only a few available studies regarding their implication for children [7, 8, 10].

Many factors were previously evaluated as predicting factors for CBD stones. While it is widely accepted that the persistent presence of a filling defect on ultrasound (US) mandates invasive investigation of the biliary tree, other indicators such as gallstone pancreatitis, bilirubin elevation, and CBD dilatation are more ambiguous as standalone predictors [11, 12]. The presence of gallstone pancreatitis was historically considered a risk factor for CBD stones. However, studies in adult patients demonstrated only a weak association between gallstone pancreatitis and the presence of CBD stones [13–15]. Further studies in pediatric patients have reported similar results, indicating that pediatric patients with gallstone pancreatitis without associated risk factors have a very low incidence of CBD stones [16]. Although conjugated bilirubin is an important factor in predicting the presence of CBD stones, the exact cutoff values warranting further evaluation in pediatric patients remain a topic of debate [7, 10]. Furthermore, some studies emphasize the importance of monitoring trends in bilirubin levels to improve the accuracy of CBD stone detection [17]. Notably, hemolytic disorders remain a relatively more common indication for cholecystectomy in children than in adults. Evaluating the risk for CBD stones in these patients using bilirubin levels is particularly challenging, as significant physiological alterations in the liver and biochemical changes due to hemolytic conditions can complicate the interpretation of bilirubin levels [10, 18]. CBD dilatation on ultrasound during initial evaluation is also considered an indicator for CBD stones. However, the exact cutoff for children is not well established, and some researchers question its validity as a sole indicator [19–21].

The 2019 American society for gastrointestinal endoscopy (ASGE) guidelines for choledocholithiasis serve as key references in the evaluation of the biliary tract in adults [11]. Patients are categorized as high, intermediate, or low risk based on clinical, laboratory, and imaging criteria. Those at high risk (above 50% probability of CBD stones) should proceed directly to endoscopic retrograde cholangiopancreatography (ERCP) for bile duct clearance. Patients with intermediate risk (10–50% probability) should undergo further non-invasive evaluation, while those at low risk (< 10% probability) can proceed to cholecystectomy without additional diagnostic procedures. This risk-based approach aims to reduce unnecessary ERCPs and their associated complications while ensuring timely intervention for those at significant risk. The most significant change in the 2019 ASGE guidelines compared to the 2010 version is the modification of the high-risk definition, which now specifies a total bilirubin level of > 4 mg/dL, but only when accompanied by a dilated bile duct on US or cross-sectional imaging. One prospective study in children evaluated this guideline for patients with suspected CBD stones who underwent ERCP, yielding relatively poor sensitivity of 20% with a specificity of 91%. However, this study did not include patients for whom ERCP was deferred and lacked longitudinal follow-up on those patients for complications due to retained stones [10].

The current study aimed to assess the incidence and predictive factors for CBD stones in children undergoing cholecystectomy for cholelithiasis, and to evaluate the applicability of adult guidelines to the pediatric population.

## Materials and methods

This retrospective study was designed to evaluate the prevalence and risk factors for CBD stones among pediatric patients undergoing cholecystectomy for cholelithiasis. The study population included children under the age of 19 who underwent cholecystectomy at a tertiary pediatric medical center from 2011 to 2024. Children with both hemolytic and non-hemolytic gallstone disease were included. Exclusion criteria for the study included patients undergoing cholecystectomy for indications other than symptomatic cholelithiasis or hemolysis-associated cholelithiasis. Demographic data, including age, gender, and comorbidities, were collected. Medical records were reviewed for clinical parameters on presentation, including laboratory and imaging findings. Elevated bilirubin was defined as a total bilirubin level greater than 4 mg/dL, with > 20% conjugated. The same cutoffs were used for both hemolytic and non-hemolytic patients.

The indication for surgery was defined as the primary reason for cholecystectomy: biliary colic, acute cholecystitis, acute cholangitis, biliary pancreatitis, choledocholithiasis, or asymptomatic cholelithiasis associated with a hemolytic disorder. Choledocholithiasis was considered the indication when patients were assigned relevant ICD-9 diagnosis codes (574.21, 574.51, or 574.91) at presentation, regardless of subsequent diagnostic confirmation.

Filling defects and dilated CBD on primary imaging were defined based on the radiologist’s interpretation. For children (up to 13 years old), the radiology team primarily relied on reference values reported by Hernanz-Schulman et al. for normal CBD diameter according to age [22]. For adolescents, a cutoff of 6 mm—consistent with adult criteria—was used. The decision to continue further evaluation of the biliary tree was based on clinical judgment and resource availability. The outcome measures included the presence of CBD stones detected on ERCP or intraoperative cholangiogram (IOC), as well as any post-cholecystectomy complications due to retained biliary stones.

Adult scoring systems were used to stratify patients based on laboratory imaging and clinical data. The ASGE guidelines classify patients as high risk for CBD stones based on the presence of clinical ascending cholangitis, visible bile duct stones on imaging (US or cross-sectional imaging), or a combination of bilirubin levels greater than 4 mg/dL and dilated CBD, intermediate risk as abnormal liver function tests or a dilated bile duct without definitive imaging evidence of stones, and low risk without any of these criteria [11].

Statistical analysis was carried out using R version 4.3.1 software (The R Foundation) and R-studio. Categorical data were reported as frequencies and percentages. Continuous variables were reported as either means and standard deviations or medians and interquartile ranges (IQR, 25th–75th percentiles), depending on their distribution. Nominal data was analyzed using the χ2 or Fisher exact tests as appropriate for the sample size. Continuous data was compared using the Mann–Whitney *U* test. A *p* value of ≤ 0.05 was considered significant. Sensitivity, specificity, positive predictive value (PPV), and negative predictive value (NPV) were calculated to evaluate the diagnostic accuracy of the ASGE high-risk criteria in this cohort. Confidence intervals (CIs) of 95% were computed for each metric to assess their precision.

## Results

A total of 177 patients diagnosed with cholelithiasis underwent cholecystectomy at a single tertiary pediatric medical center during the years 2011–2024. The median age was 13.4 years (IQR 9,16.4). The most common indication for surgery was biliary colic, involving 97 patients (55%). Complicated diseases, including choledocholithiasis, gallstone pancreatitis, and cholangitis, were observed in 37 (21%) patients.

The majority of patients, 150 (84.7%), underwent cholecystectomy without additional biliary evaluation and experienced no readmissions for complications related to retained stones. Sixteen patients (9%) were found to have CBD stones, confirmed by either ERCP or IOC, or through post-cholecystectomy complications indicative of retained stones. An additional 11 patients (6.2%) underwent further biliary tree evaluation but showed no evidence of CBD stones. The median ages of the CBD stones group and the non-CBD stones group were similar (13.2 vs. 13.4, *p* = 0.47) (Table 1), with the youngest patient presenting with CBD stones being 2.3 years old. The presence of elevated bilirubin, dilated CBD, and filling defects on primary imaging was significantly higher in the CBD stones group (50.0% vs. 9.9%, *p* < 0.001, 62.5% vs. 9.3%, *p* < 0.001, and 43.8% vs. 4.4%, *p* < 0.001, respectively). The rate of gallstone pancreatitis was also significantly higher in the CBD stones group (25% vs. 6.8%, *p* = 0.04). However, all patients with gallstone pancreatitis who were ultimately diagnosed with CBD stones also exhibited other high-risk predictors, such as filling defects, dilated common bile ducts on ultrasound, or elevated bilirubin levels.

**Table 1:** Characteristics of pediatric patients with and without CBD stones

Characteristic	CBD stones (*n* = 16)	No CBD stones (*n* = 161)	*p* Value
Median age in years (IQR)	13.25 (8.1–14.6)	13.40 (9.0–16.4)	0.48
Male gender	3 (18.8%)	52 (32.3%)	0.40
Overweight or obese	4 (25.0%)	49 (30.4%)	0.87
Hemolytic disorder	7 (43.8%)	56 (34.8%)	0.66
Gallstone pancreatitis	4 (25.0%)	11 (6.8%)	0.04
Abnormal liver function tests	13 (81.3%)	36 (22.4%)	< 0.001
Elevated bilirubin^a^	8 (50.0%)	16 (9.9%)	< 0.001
Dilated CBD on ultrasound	10 (62.5%)	15 (9.3%)	< 0.001
Filling defects on ultrasound	7 (43.8%)	7 (4.4%)	< 0.001

*IQR* Interquartile range; *CBD* Common bile duct

a Total bilirubin > 4 mg/dL and direct bilirubin > 20%

Overall, 63 patients (35.6%) had a hemolytic disorder. There was no significant difference in the prevalence of hemolytic disorders between children with CBD stones and those without (43.8% vs. 34.8%, *p* = 0.66). Twenty-eight patients (15.8%) underwent cholecystectomy for asymptomatic cholelithiasis with or without concurrent splenectomy. Their median preoperative bilirubin level was 2.67 (IQR 1.9,4.2) and their median conjugated bilirubin level was 0.6 (IQR 0.5,0.7). None of these patients exhibited bilirubin levels meeting the defined cutoff. Of the 35 patients with hemolysis-related symptomatic cholelithiasis, 7 had CBD stones (20%), and 6 of these had bilirubin elevation.

According to the ASGE guidelines, 21 patients (11.9%) were classified as high risk, and 31 (17.5%) as intermediate risk (Table 2). In the high-risk group, preoperative MRCP was performed on 7 (33.3%) patients, revealing CBD filling defects in only 1 (4.8%). Preoperative ERCP was conducted on 8 patients (38.1%), with stones identified in 6 (28.6%). Postoperatively, ERCP was necessary for 2 (9.5%) patients, with stones found in 1 (4.8%). The readmission rate for complications due to retained stones in the high-risk group was 9.5%, and the overall incidence of CBD stones was 42.8%. In the intermediate-risk group, preoperative MRCP was performed on 3 (6.5%) patients, though none showed filling defects. Preoperative ERCP was conducted on 4 (12.9%) patients, with stones detected in 3 (9.7%). Postoperatively, ERCP was necessary for 2 (6.5%) patients, with stones found in 1 (3.2%). The readmission rate was 6.5%, and the overall incidence of CBD stones was 16.1%. For low-risk patients, only 1 underwent MRCP, which revealed no evidence of CBD stones. The readmission rate for CBD stones and the overall CBD stones rate were both 1.6%. Overall, only three patients in this cohort underwent IOC, with CBD stones detected in only one.

**Table 2:** Management and outcomes by ASGE risk group in pediatric patients with cholelithiasis

Characteristic	High risk (*n* = 21)	Intermediate risk (*n* = 31)	Low risk (*n* = 125)
Pre-Op MRCP	7 (33.3%)	3 (6.5%)	1 (0.8%)
Filling defects on MRCP	1 (4.8%)	0 (0%)	0 (0%)
Pre-Op ERCP	8 (38.1%)	4 (12.9%)	0 (0%)
Stones on pre-Op ERCP	6 (28.6%)	3 (9.7%)	0 (0%)
Post-Op ERCP	2 (9.5%)	2 (6.5%)	0 (0%)
Stones on post-Op ERCP	1 (4.8%)	1 (3.2%)	0 (0%)
IOC	2 (9.5%)	1 (3.2%)	0 (0%)
Stones on IOC	1(4.8%)	0 (0%)	0 (0%)
Readmission for complications due to retained CBD stones	2 (9.5%)	2 (6.5%)	2 (1.6%)
Overall CBD stones	9 (42.8%)	5 (16.1%)	2 (1.6%)

*MRCP* Magnetic resonance cholangiopancreatography; *ERCP* Endoscopic retrograde cholangiopancreatography; *IOC* Intraoperative cholangiography; *CBD* Common bile duct

The ASGE guidelines were assessed for their diagnostic performance in detecting CBD stones among high-risk pediatric patients within this cohort (Table 3). The sensitivity of the guidelines was recorded at 56.2% (95% CI 29.9–80.2%), and specificity was noted at 91.9% (95% CI 86.6–95.6%). The positive predictive value (PPV) and negative predictive value (NPV) were 40.9% (95% CI 20.7–63.6%) and 95.5% (95% CI 90.9–98.2%), respectively. Notably, modifying the ASGE guidelines to include patients with elevated bilirubin as high risk improved the sensitivity to 68.8% (95% CI 41.3–89.0%), with the specificity slightly reduced to 87.6% (95% CI 81.5–92.2%).

**Table 3:** Diagnostic performance of ASGE guidelines for detecting CBD stones in pediatric patients

Measure	ASGE	Modified ASGE^a^
Sensitivity % (95% CI)	56.2% (29.9–80.2%)	68.8% (41.3–89.0%)
Specificity % (95% CI)	91.9% (86.6–95.6%)	87.6% (81.5–92.2%)
Positive predictive value % (95% CI)	40.9% (20.7–63.6%)	35.5% (19.2–54.6%)
Negative predictive value % (95% CI)	95.5% (90.9–98.2%)	96.6% (92.2–98.9%)

a High-risk criteria were modified to include patients with an isolated finding of elevated bilirubin

Within the subgroup with hemolytic disorders, the sensitivity of the original ASGE guidelines was 85.7% (95% CI 42.1–99.6%), and the specificity was 89.3% (95% CI 78.1–96.0%). After modifying the scoring criteria, sensitivity remained 85.7% (95% CI 42.1–99.6%) and specificity decreased to 82.1% (95% CI 69.6–91.1%).

## Discussion

The prevalence of cholelithiasis necessitating cholecystectomy in children is on the rise. However, the perioperative evaluation for CBD stones in pediatric patients undergoing cholecystectomy relies primarily on guidelines developed for adults. Previous studies have highlighted that the sensitivity of these adult guidelines is relatively low when applied to the pediatric population, emphasizing the need for pediatric-specific guidelines [10]. Although several scoring systems have been proposed to predict CBD stones in children [8, 18], there are currently no structured guidelines specifically tailored for risk stratification and management of pediatric patients undergoing cholecystectomy for cholelithiasis.

Our results show that common bile duct stones complicate cholelithiasis in 20% of pediatric patients, presenting as gallstone pancreatitis, cholangitis, or choledocholithiasis. This prevalence aligns with previous findings reported in the literature [23]. However, the rate of true CBD stones mandating CBD clearance is probably closer to 10%. As in previous studies involving both children and adults, elevated bilirubin, filling defects, and dilated CBD observed on ultrasound were highly associated with CBD stones. However, it is challenging to determine which risk factors can independently predict CBD stones and which are clinically significant only when accompanied by other factors, as all these indicators are highly interrelated and represent the same pathophysiological condition. Regarding filling defects on US, it is widely agreed that these defects mandate, in adults and in children, a perioperative attempt at CBD clearance. Our findings also support this approach. However, although filling defects carry a high positive predictive value, they are described as particularly insensitive and thus serve as a poor screening tool [18]. In this cohort, out of 16 children who were eventually diagnosed with CBD stones, less than half presented with a filling defect on primary imaging (7 children, 44%). Regarding CBD dilation, although it is highly associated with the presence of CBD stones, some previous literature casts doubt on its value as a solitary predictor when liver function tests (LFTs) and bilirubin are normal [21]. While for adults the CBD cutoff is more unequivocal, applying these adult cutoffs to children results in less than 50% detection of CBD stones. In children, there is variability in CBD diameter, and the true cutoff for pathologic CBD is not well established and likely varies with age [24]. Moreover, while adults presenting with solitary CBD dilatation typically require further assessment, ampullary tumors are extremely rare in children, and so these patients might not need any further evaluation when CBD stones are excluded [25].

In this study, we addressed elevated bilirubin levels, defined as total bilirubin > 4 mg/dL and direct bilirubin > 20%, among both hemolytic and non-hemolytic patients. While other studies have excluded hemolytic patients due to variations in liver function tests and bilirubin levels, we thought it important to maintain a structured approach that is also applicable to children with hemolytic disorders. Thus, we incorporated the standard cutoff for conjugated bilirubin into the ASGE criteria, ensuring that only children with true conjugated hyperbilirubinemia were classified as high risk. Notably, none of the children with asymptomatic hemolysis-related cholelithiasis met the defined bilirubin cutoff. This finding supports our conclusion, as it suggests that this cutoff effectively distinguishes between bilirubin elevation caused by hemolysis and bilirubin elevation resulting from complicated cholelithiasis. It is important to note that we did not examine any bilirubin cutoffs other than those specified in the ASGE guidelines. However, we believe that a larger cohort may be necessary to establish precise bilirubin cutoffs for children, which might require adjustment based on age or body measurements.

As no single reliable predictor for CBD stones exists, any protocol for biliary tree clearance should aim to balance sensitivity and specificity to minimize the risks of false-positive and false-negative cases. Overestimating the risk for CBD stones may lead to unnecessary preoperative ERCP, exposing patients to potential complications such as pancreatitis, bleeding, and anesthesia-related adverse events. Conversely, underestimating the risk may delay necessary intervention for CBD stones, potentially resulting in postoperative cholangitis, pancreatitis, or the need for urgent ERCP.

According to this cohort, the sensitivity, specificity, PPV, and NPV of the ASGE guidelines were recorded at 56.2, 91.9, 40.9 and 95.5%, respectively. Several validation studies demonstrated a similar sensitivity of 37–68% and a specificity of 52–80% for the presence of CBD stones in ASGE high-risk adults [26–29]. A study applying ASGE criteria to children undergoing ERCP demonstrated a relatively low sensitivity of 20% with a specificity of 91% for high-risk criteria. [10]. However, it is difficult to generalize these results to all children with cholelithiasis, as those who underwent ERCP represent a selected subgroup likely already having a high prevalence of high-risk predictors. When adding elevated bilirubin as a solitary predictor in our cohort, the sensitivity rose to 68.8%, with specificity of 87.6%. Based on this finding, we think it is reasonable to perform upfront preoperative ERCP for children with a consistent bilirubin elevation of > 4 mg/dl with > 20% conjugated without a confirmed CBD dilatation. Given that cutoffs for CBD diameter in children are less established than in adults, we believe it is unwise to mandate invasive evaluation of the biliary tree in CBD dilatation. Regarding children with hemolytic disorders, the ASGE guidelines demonstrated very good predictive value for this patient group. Notably, modifying the guidelines to include solitary bilirubin elevation as a high-risk feature reduced specificity without affecting sensitivity; however, both remained relatively high.

In our cohort, only three patients underwent IOC. This was primarily due to the cohort’s relatively older data range starting from 2011 and the higher availability of ERCP services at our institution. Nevertheless, we think it is necessary to address recent publications in pediatric patients suggesting that IOC with a laparoscopic common bile duct exploration (LCBDE) as necessary could replace most preoperative ERCP procedures. This approach has been shown to reduce hospitalization time and time to definitive intervention, while also resulting in fewer procedures [30–32]. It is hard to evaluate post hoc the probability of a successful ‘surgery-first’ approach in this cohort, specifically due to the lack of data regarding the size of stones extracted during ERCP. Nevertheless, based on previous data, we do believe it is reasonable to suggest IOC ± LCBDE for pediatric patients with high-risk features on preoperative evaluation in experienced institutions where this option is highly available.

**Fig. 1:**
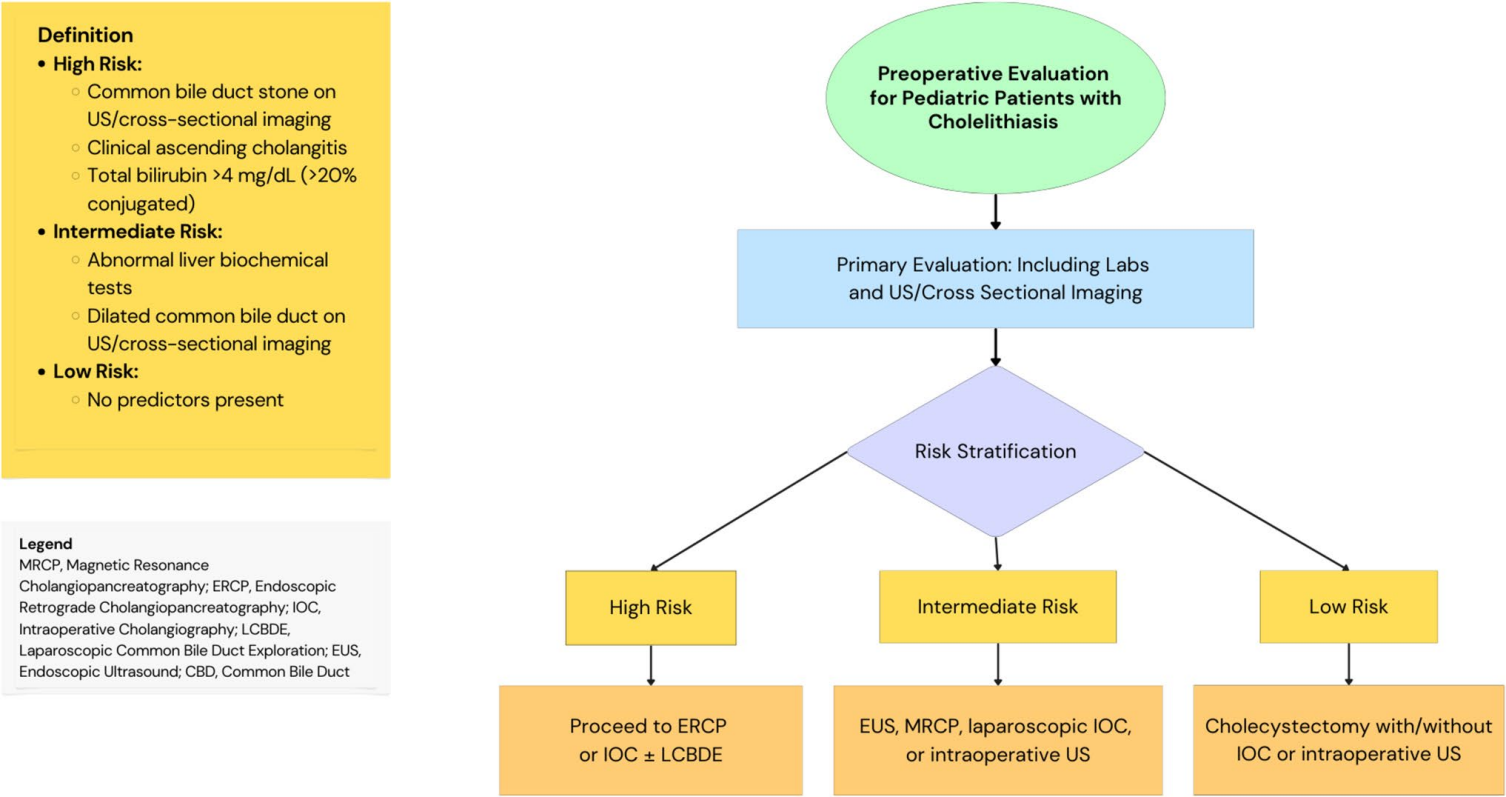
Proposed strategy to assign risk of CBD stones and manage pediatric patients undergoing cholecystectomy for cholelithiasis based on clinical predictors

Based on our findings and previously published data regarding the prevalence and risk factors for CBD stones in pediatric patients, we suggest this algorithm for the perioperative evaluation of CBD stones in pediatric patients prior to cholecystectomy (Fig. 1).

This study’s limitations revolve around its retrospective nature and single-center experience. The decision to continue further evaluation of the biliary tree was based on clinical judgment and resource availability; thus, not all patients underwent the same evaluation. Moreover, quantifying the true rate of CBD stones necessitating bile duct clearance is challenging, as biliary stones are probably a more dynamic condition: some of the stones evacuated during ERCP might prove to be clinically insignificant post-cholecystectomy. Additionally, some postoperative biliary stones might have migrated to the common bile duct during surgery and do not represent true preoperative CBD stones. However, we recognize that a positive preoperative ERCP is considered justified, and that the presence of postoperative evidence of choledocholithiasis may suggest a preoperative retained stone. Nevertheless, while other studies lack follow-up for patients who undergo cholecystectomy without any further workout, we retrospectively screened all files. This allowed us to accurately quantify the true rate of complications due to retained stones in these patients. Further prospective studies with larger cohorts are necessary to properly evaluate the algorithm for pediatric preoperative biliary clearance suggested in this paper.

## Data Availability

No datasets were generated or analysed during the current study.
